# Single-cell transcriptomics enable the characterization of local extension in retinoblastoma

**DOI:** 10.1038/s42003-023-05732-y

**Published:** 2024-01-03

**Authors:** Yaoming Liu, Wei Hu, Yanjie Xie, Junjie Tang, Huan Ma, Jinmiao Li, Jiahe Nie, Yinghao Wang, Yang Gao, Chao Cheng, Cheng Li, Yujun Ma, Shicai Su, Zhihui Zhang, Yuekun Bao, Yi Ren, Xinyue Wang, Fengyu Sun, Shengli Li, Rong Lu

**Affiliations:** 1 State Key Laboratory of Ophthalmology, Zhongshan Ophthalmic Center, Sun Yat-sen University, Guangdong Provincial Key Laboratory of Ophthalmology and Visual Science, 510060 Guangzhou, China; 2 Precision Research Center for Refractory Diseases, Institute for Clinical Research, Shanghai General Hospital, Shanghai Jiao Tong University School of Medicine, 201620 Shanghai, China

**Keywords:** Data mining, Eye cancer

## Abstract

Retinoblastoma (RB) is the most prevalent ocular tumor of childhood, and its extraocular invasion significantly increases the risk of metastasis. Nevertheless, a single-cell characterization of RB local extension has been lacking. Here, we perform single-cell RNA sequencing on four RB samples (two from intraocular and two from extraocular RB patients), and integrate public datasets of five normal retina samples, four intraocular samples, and three extraocular RB samples to characterize RB local extension at the single-cell level. A total of 128,454 qualified cells are obtained in nine major cell types. Copy number variation inference reveals chromosome 6p amplification in cells derived from extraocular RB samples. In cellular heterogeneity analysis, we identified 10, 8, and 7 cell subpopulations in cone precursor like cells, retinoma like cells, and MKI67^+^ photoreceptorness decreased (MKI67^+^ PhrD) cells, respectively. A high expression level of *SOX4* was detected in cells from extraocular samples, especially in MKI67^+^ PhrD cells, which was verified in additional clinical RB samples. These results suggest that *SOX4* might drive RB local extension. Our study presents a single-cell transcriptomic landscape of intraocular and extraocular RB samples, improving our understanding of RB local extension at the single-cell resolution and providing potential therapeutic targets for RB patients.

## Abstract (web summary)

Single-cell transcriptomic characterization of intraocular and extraocular retinoblastoma suggests that SOX4 may drive the local extension of retinoblastoma.

## Introduction

Retinoblastoma (RB) is the most common eye cancer of childhood. Its prognosis has improved over the past 50 years^1,2^, and RB has become a curable disease in high-income countries, with a ten-year overall survival rate greater than 95%^3^. Attention has now shifted to eye salvage^4^ and improvement of life quality. However, the current salvage rate of RB eyes is still unsatisfactory. Even in high-income countries, 30% of patients (mostly in advanced intraocular stage) initially or eventually undergo enucleation^5^. Besides, in low- and middle-income countries, where more than 80% of global RB cases arise, the prognosis is poor due to delayed diagnosis and treatment^6–8^. Appropriately 49.1% of patients from low-income countries have extraocular RB, and 18.9% of cases have metastatic progression^3^.

A drastic increase in the risk of RB metastasis is associated with increased tumor volume and the presence of extraocular disease^9^. RB patients with extraocular tumors involving the retrobulbar optic nerve or the orbit suffer a dramatically increased risk (greater than 48.55-fold) of metastasis compared to those with intraocular tumors, and the cumulative survival proportion shows a steep decline from 100% to 45%^9^. Locally advanced RB is more life-threatening, making eye salvage less likely. Whether to choose eye enucleation or eye salvage becomes a significant dilemma for patients’ family and doctors. Generally, the steps in the metastatic process involve the initiation of local extension and metastasis, followed by migration towards distant metastatic sites, and subsequent colonization^10^. Clearly, the prognosis of RB patients worsens drastically once RB breaches the ocular coats and develops local extension and invasion^9^. However, the mechanism of extraocular extension of RB has not yet been elucidated. A better understanding of the molecular characteristics that give rise to metastatic cells during the local extension step of RB metastasis will lead to better strategies for patient treatment and outcomes.

Bulk RNA-seq studies provide general transcriptomic information for the entire tissue sample but overlook the intra-tumoral heterogeneity of RB. Recent advances in single-cell technology are facilitating a deeper understanding of tumor cell heterogeneity in the metastasis process^11,12^. Single-cell RNA sequencing (scRNA-seq) has been applied to identify the diversity of cell types in ocular tumors^13,14^. However, there has been no study comparatively investigating tumor cell heterogeneity and the molecular characteristics that give rise to the local extension of RB and metastasis. The core cell subpopulations and genes contributing to the local extension of RB remain unknown. Our study applied scRNA-seq to both intraocular and extraocular RB (involving the retrobulbar optic nerve) to comprehensively profile cellular and molecular signature regarding local extension progression. This will provide insight into the mechanisms underlying RB progression.

## Results

### Single-cell characterization of transcriptional landscape in intraocular and extraocular RB

**Fig. 1:**
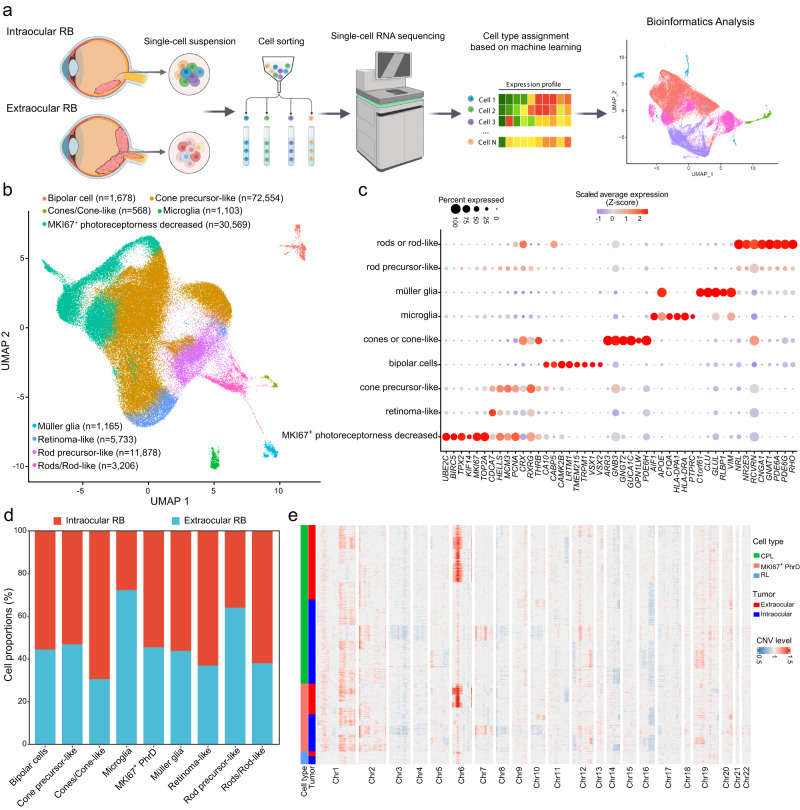
Single-cell transcriptomics in RB. **a** Overall design schematic of the study. The cartoon was created with BioRender.com **b** UMAP dimensionality reduction of all qualified cells. **c** Representative gene markers identified for each cell type. **d** Proportions of cells derived from intraocular and extraocular RB samples in each cell type. **e** Inferred copy number variations (CNVs) of CPL, RL, and MKI67^+^ PhrD cells in intraocular and extraocular RB samples across 22 chromosomes.

To characterize the transcriptional landscape of intraocular and extraocular RB at the single-cell level, we applied scRNA-seq to four RB samples (two from intraocular RB patients and two from extraocular RB patients) and integrated public scRNA-seq datasets^15,16^ of five normal retina samples, four intraocular, and three extraocular RB samples (Fig. 1a). The scRNA-seq data underwent quality control, batch effects correction, normalization, integration, and clustering by employing the Seurat R package (see Methods). The data integration removed batch effects among different samples (Supplementary Fig. 1a and 1b). A total of 128,454 qualified cells were obtained after the removal of low-quality cells and doublets. In total, 55,492 and 61,882 cells were detected in extraocular and intraocular RB samples, respectively. A portion of cells was exclusively detected in extraocular RB samples, potentially indicating a high-level capability of local extension (Supplementary Fig. 1c). Cell cycle phase analysis revealed separate cell clusters with different phases (Supplementary Fig. 1d). The effect of the cell cycle was regressed out before clustering. All cells were clustered into 24 clusters based on their expression patterns of highly variable genes (Supplementary Fig. 1e). Based on the expression features of curated cell type markers (see Methods), nine different cell types were identified among these cell clusters, including cone precursor-like cells (CPL cells, *n* = 72,554), MKI67^+^ photoreceptorness decreased cells (MKI67^+^ PhrD cells, *n* = 30,569), rod precursor-like cells (*n* = 11,878), retinoma-like cells (RL cells, *n* = 5,733), rods or rod-like cells (*n* = 3206), bipolar cells (*n* = 1678), Müller glia (*n* = 1165), microglia (*n* = 1103), and cones or cone-like cells (*n* = 568) (Fig. 1b). These cell types showed exclusive expression of corresponding cell type markers, indicating featured biological functions (Fig. 1c and Supplementary Data 1). For example, MKI67^+^ PhrD cells expressed high levels of the *MKI67*, *TOP2A*, *UBE2C*, *BIRC5*, and *TPX2* genes (Fig. 1c). In this dataset, cones or cone-like, rods or rod-like, and retinoma-like cells were mainly found in intraocular RB samples, while microglia and rod precursor-like cells were mostly detected in extraocular RB samples (Fig. 1d). Moreover, gains at chromosomal arms 1q, 2p, and 6p and losses at 3q and 16q were detected in cells of RB tumors. Intriguingly, extraocular RB samples had a larger proportion of cells (52.8%) with CNV gains in chromosome 6p than intraocular RB samples (Fig. 1e). We found that 80% (4/5) of extraocular RB samples but none of intraocular RB samples showed remarkable chromosome 6p amplification (Supplementary Fig. 2a–c). Extraocular RB samples exhibited higher overall CNV levels than intraocular RB samples (Supplementary Fig. 2d–f). Collectively, our analysis presented cellular and molecular discrepancies between intraocular and extraocular RB samples through single-cell transcriptional profiling.

### Transcriptional disturbances in cone precursor-like, retinoma-like, and MKI67^+^ PhrD cells

**Fig. 2:**
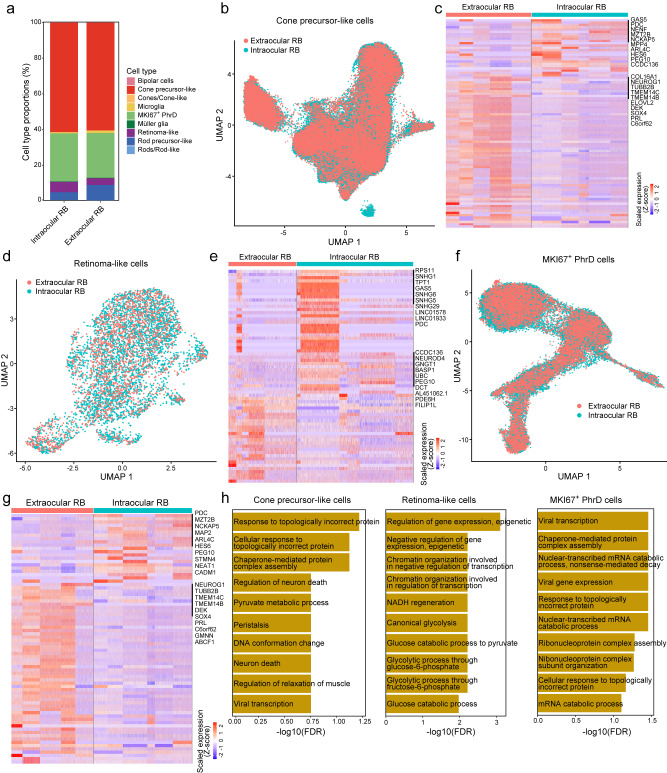
Differences between intraocular and extraocular RB samples for each cell type. **a** Proportions of different cell types in intraocular and extraocular RB samples. **b** UMAP plot displaying the cone precursor-like cells in intraocular and extraocular RB samples. **c** Heatmap depicting the differentially expressed genes (DEGs) in cone precursor-like cells between intraocular and extraocular RB samples. **d** UMAP plot illustrating retinoma-like cells in intraocular and extraocular RB samples. **e** Heatmap presenting the DEGs in retinoma-like cells between intraocular and extraocular RB samples. **f** UMAP plot showing the MKI67^+^ PhrD cells in intraocular and extraocular RB samples. **g** Heatmap displaying the DEGs in MKI67^+^ PhrD cells between intraocular and extraocular RB samples. **h** Functional enrichment of DEGs in cone precursor-like cells, retinoma-like cells, and MKI67^+^ PhrD cells.

Cone precursor-like cells, retinoma-like cells, and MKI67^+^ PhrD cells constituted the major cell components of both intraocular and extraocular RB samples (Fig. 2a). The cell proportions of these three cell types showed no significant differences between intraocular and extraocular RB samples. The majority of CPL cells exhibited similar overall expression patterns between intraocular and extraocular RB samples (Fig. 2b). However, individual genes exhibited differential expression levels, wherein 66 genes were upregulated, such as *TUBB2B* and *TMEM14C*, and 25 genes were downregulated in extraocular RB samples, such as *GAS5* and *PDC* (Fig. 2c and Supplementary Data 2). Regarding RL cells, a similar portion was detected between intraocular and extraocular RB samples (Fig. 2d). In extraocular RB samples, 27 genes were upregulated and 40 genes were downregulated in RL cells (Fig. 2e and Supplementary Data 2). Subsets of MKI67^+^ PhrD cells were also well-distributed in extraocular and intraocular RB samples (Fig. 2f). In MKI67^+^ PhrD cells, 55 genes showed upregulation, and 21 genes showed downregulation in extraocular RB samples (Fig. 2g and Supplementary Data 2). Furthermore, enrichment analyses were performed to investigate the potential differences in biological functions between intraocular and extraocular RB samples. Differential genes in CPL and MKI67^+^ PhrD cells showed enrichment in protein stability-related functions, such as “Protein stability”, “Response to unfolded protein”, and “Response to topologically incorrect protein” (Fig. 2h). Besides, RL cells showed differences in metabolism-related functions, such as “Canonical glycolysis” and “NADH regeneration”. In summary, we characterized cell type-specific transcriptional differences between intraocular and extraocular RB in CPL, RL, and MKI67^+^ PhrD cells.

### Delineation of cellular heterogeneity in major cell types of RB samples

**Fig. 3:**
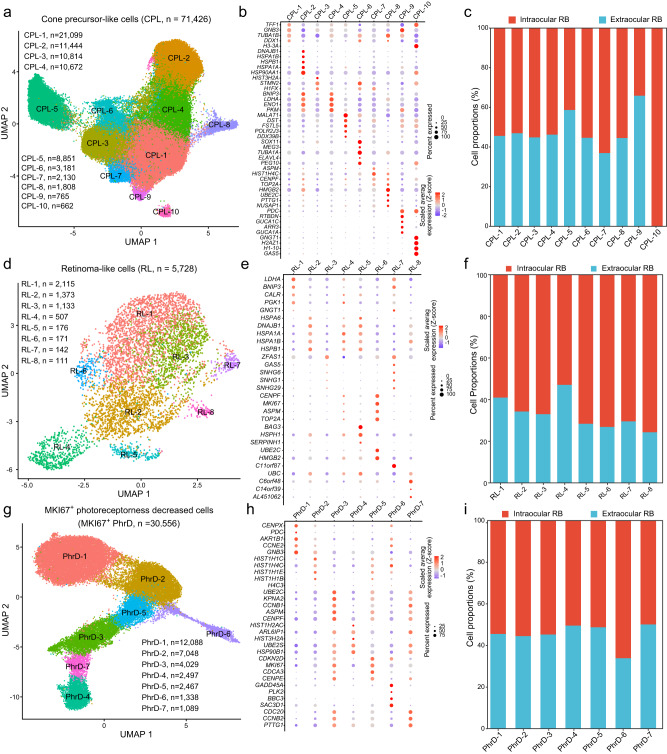
Subpopulations of cone precursor-like, retinoma-like, and MKI67^+^ PhrD cells in RB. **a** Ten subpopulations were identified in CPL cells. **b** The relative expression of top marker genes of each CPL subpopulation across all subpopulations. **c** The proportions of different CPL subpopulations in intraocular and extraocular RB samples. **d** Eight subpopulations were identified in RL cells. **e** The relative expression of top marker genes of each RL cell subpopulation across all subpopulations. **f** The proportions of different RL cell subpopulations in intraocular and extraocular RB samples. **g** Seven subpopulations were identified in MKI67^+^ PhrD cells. **h** The relative expression of top marker genes of each MKI67^+^ PhrD cell subpopulation across all subpopulations. **i** The proportions of different MKI67^+^ PhrD cell subpopulations in intraocular and extraocular RB samples.

To further explore cellular heterogeneity in CPL, RL, and MKI67^+^ PhrD cells, we identified subpopulations in each cell type. Ten cell subpopulations were detected in CPL (Fig. 3a). These 10 CPL subpopulations were characterized by different gene markers (Fig. 3b and Supplementary Data 3). The largest CPL subpopulation (CPL-1, n = 21,099) showed exclusive expression of *TFF1* and *TUBA1B*. The CPL-1 subpopulation exhibited energy metabolism-related processes, such as “Mitochondrial respiratory chain complex assembly” (Supplementary Fig. 3a). The CPL-7 and CPL-10 subpopulations were mainly detected in intraocular RB samples, while most portions of the CPL-5 and CPL-9 cell subpopulations were found in extraocular RB samples (Fig. 3c). In the 5,733 RL cells, we distinguished 8 RL cell subpopulations (Fig. 3d). The RL-4, RL-5, RL-7, and RL-8 cells showed highly distinct transcriptomic features from other subpopulations. The RL-5 subpopulation was featured with high expression of genes *BAG3* and *HSPH1*, while the RL-7 showed exclusive expression of gene *C11or87* (Fig. 3e and Supplementary Data 3). The RL-3, RL-7, and RL-8 subpopulations highly expressed endoplasmic reticulum-related genes (Supplementary Fig. 3b). Most portions of the RL subpopulations were in intraocular RB samples (Fig. 3f). MKI67^+^ PhrD cells were divided into 7 different subpopulations based on clustering from gene expression profiles (Fig. 3g). The PhrD-1 and PhrD-2 subpopulations constituted a large proportion (62.6%) of MKI67^+^ PhrD cells. The PhrD-1 subpopulation was characterized by high expression of genes *CENPX* and *GNB3*, whereas the PhrD-3 subpopulation expressed high levels of genes *ASPM* and *UBE2C* (Fig. 3h and Supplementary Data 3). The PhrD-2 subpopulations showed high activity in cell proliferation-related functions, such as “Cell cycle G1/S phase transition” and “Nuclear division” (Supplementary Fig. 3c). The PhrD-6 cell subpopulations were mainly detected in intraocular RB samples (Fig. 3i). In summary, our cell subcluster analysis revealed cellular and molecular heterogeneity in CPL, RL, and MKI67^+^ PhrD cells of RB samples.

### Reconstruction of transcriptional trajectory in intraocular and extraocular RB

**Fig. 4:**
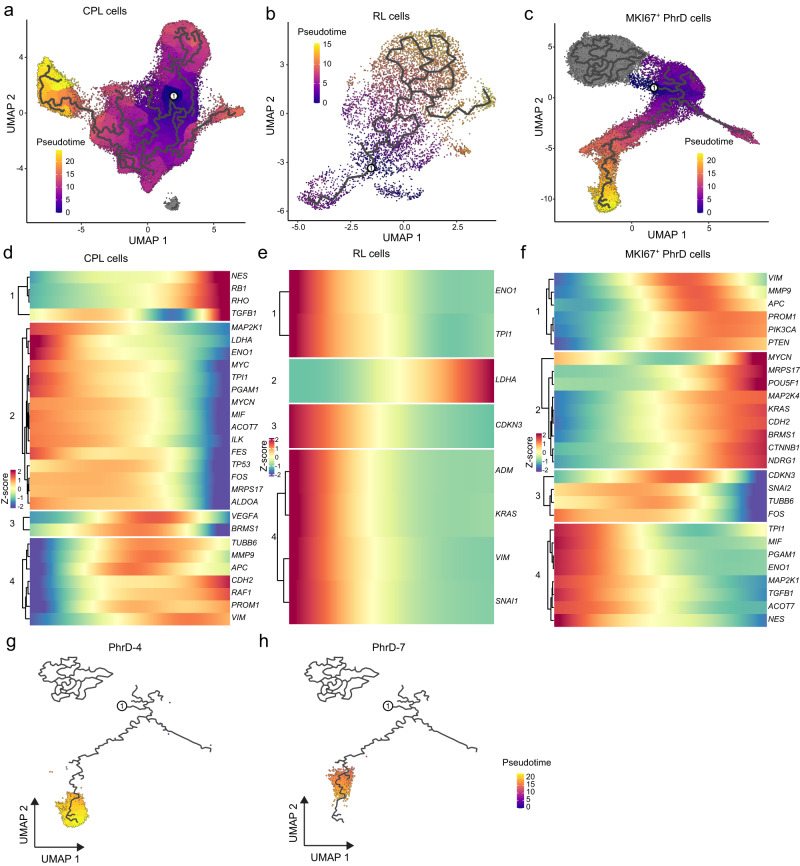
Pseudo-time trajectory analysis of CPL cells, RL cells, and MKI67^+^ PhrD cells. **a** Pseudo-time trajectory of CPL cells in intraocular and extraocular RB samples. **b** Pseudo-time trajectory of RL cells in intraocular and extraocular RB samples. **c** Pseudo-time trajectory of MKI67^+^ PhrD cells in intraocular and extraocular RB samples. **d** Heatmap showing the four clustering patterns of DEGs along the pseudo-time path of CPL cells. **e** Heatmap showing the four clustering patterns of DEGs along the pseudo-time path of RL cells. **f** Heatmap presenting the four clustering patterns of DEGs along the pseudo-time path of MKI67^+^ PhrD cells. **g** Pseudo-time trajectory of the PhrD-4 subpopulation. **h** Pseudo-time trajectory of the PhrD-7 subpopulation.

Next, we performed pseudo-time trajectory analysis to infer the evolution process of major cell types and their subpopulations during the development of local extension. The CPL, RL, and MKI67^+^ PhrD cells all showed a clear pseudo-time path (Fig. 4a–c). The observation suggested that some subsets of these cells developed local extension. These cell subpopulations also showed high G2M scores (Supplementary Fig. 4a–c). Four clusters with different expression patterns were identified in CPL, RL, and MKI67^+^ PhrD cells (Fig. 4d–f). Genes in Cluster 1 of CPL cells, such as *NES*, *RB1*, and *RHO*, showed exclusive high expression at the end of the pseudo-time trajectory (Fig. 4d). Cluster 1 genes might endow the local extension of some CPL cells. In RL cells, Clusters 1, 3, and 4 showed high transcriptional activity at the beginning, while Cluster 2 exhibited high expression only at the end of the pseudo-time trajectory (Fig. 4e). Cluster 4 of MKI67^+^ PhrD cells showed decreasing expression levels along the pseudo-time trajectory (Fig. 4f). Genes in Cluster 2 of MKI67^+^ PhrD cells showed increasing expression along the pseudo-time trajectory, such as *MYCN* and *POU5F1*, indicating their roles in promoting local extension in RB. The pseudo-time trajectories of cell subpopulations in CPL, RL, and MKI67^+^ PhrD cells were further analyzed. Cells in the CPL-5 subpopulations distributed at the end of the CPL trajectory path (Supplementary Fig. 4d). At the end of the pseudo-time trajectory path, only a small proportion of RL cells (*i.e*. the RL-7 and RL-8 subpopulations) were detected (Supplementary Fig. 4e). For the MKI67^+^ PhrD cells, the PhrD-2 subpopulation was detected at the beginning of the trajectory path, while the PhrD-5 and PhrD-6 cells distributed in the middle of the pseudo-time path (Supplementary Fig. 4f). The major portions of the MKI67^+^ PhrD-4 and PhrD-7 subpopulations were detected at the end of the trajectory (Figs. 4g, h). These MKI67^+^ PhrD cell subpopulations may develop at the end of the progression of RB extension to extraocular sites.

### Regulatory network inference identified *SOX4* as a candidate driver in the local extension of RB

**Fig. 5:**
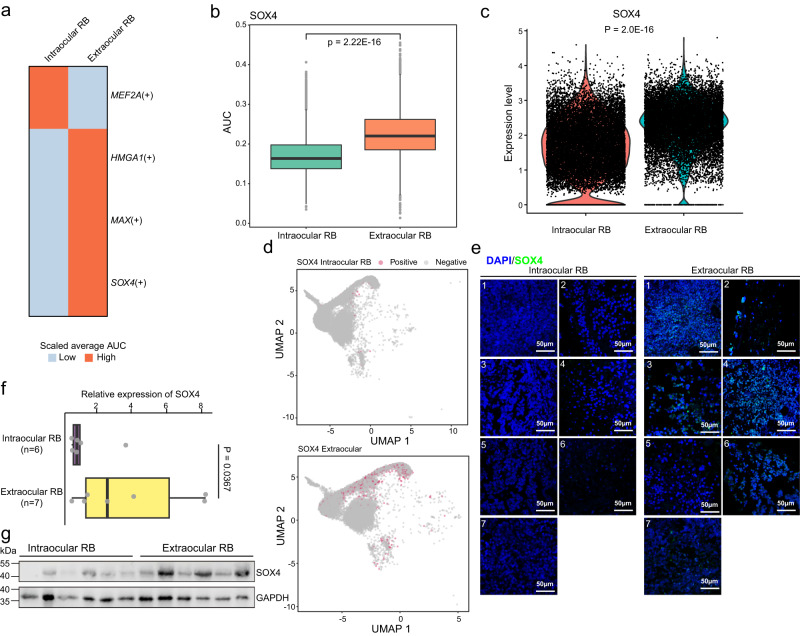
SCENIC analysis and expression validation of SOX4 in intraocular and extraocular RB samples. **a** Bi-color heatmap showing the relative AUC values of key transcription factors in intraocular and extraocular RB samples. **b** Boxplot showing the comparison of AUC values of *SOX4* in cells derived from intraocular and extraocular RB samples. Each box represents the IQR and median of AUC values in each sample group, whiskers indicate 1.5 times IQR. P, Wilcoxon’s rank-sum test. *n* = 13,911 cells in extraocular RB samples, *n* = 16,645 cells in intraocular RB samples. **c** Comparison of *SOX4* expression between cells derived from intraocular and extraocular RB samples. P, Wilcoxon’s rank-sum test. *n* = 13,911 cells in extraocular RB samples, *n* = 16,645 cells in intraocular RB samples. **d** UMAP plots highlighting the binary activity of *SOX4* in MKI67^+^ PhrD cells of intraocular (upper panel) and extraocular (bottom panel) RB samples. **e** Immunofluorescence of *SOX4* in intraocular (*n* = 7) and extraocular (*n* = 7) patient samples. **f** Comparison of *SOX4* expression between intraocular (*n* = 6) and extraocular (*n* = 7) RB samples by RT-qPCR. P, Wilcoxon rank-sum test (the alternative hypothesis is set as “greater”). Each box represents the IQR and median of SOX4 expression in each sample group, whiskers indicate 1.5 times IQR. **g** Western blot analysis of *SOX4* in intraocular (*n* = 6) and extraocular (*n* = 6) RB samples.

To identify transcription factors that may participate in the regulation of local extension in RB, we adopted the single-cell regulatory network inference and clustering (SCENIC) method^17^. Our inference analysis revealed one and three specific transcription factors that showed significant regulatory roles in intraocular and extraocular RB samples, respectively (Fig. 5a). Among them, *SOX4* exhibited a remarkably higher AUC value in extraocular RB samples (Fig. 5b). *SOX4* was highly expressed in the cells from extraocular RB samples compared to those from intraocular RB samples (Fig. 5c). In addition, the regulatory activity of *SOX4* was detected in more MKI67^+^ PhrD cells of extraocular RB samples (Fig. 5d).

Furthermore, we conducted immunofluorescence of *SOX4* in 7 clinical samples diagnosed with intraocular RB and 7 samples diagnosed with extraocular RB. Our analysis showed that *SOX4* was highly expressed in extraocular RB samples (Fig. 5e). Our qPCR (Fig. 5f) and western blot (Fig. 5g and Supplementary Fig. 7) assays further exhibited that *SOX4* was highly expressed in extraocular RB samples compared to intraocular RB samples. In addition, our correlation analysis revealed that the *SOX4* gene expression level was significantly positively correlated with inferred CNV levels in CPL cells, RL cells, and MKI67^+^ PhrD cells, respectively (Supplementary Fig. 5).

To further explore the relationship between SOX4 expression and clinicopathological features of RB development and progression, we performed SOX4 immunostaining in a cohort of 47 RB tumor samples. This cohort encompassed 22 male individuals (46.8%) and 25 female individuals (53.2%). By calculating immunoreactivity scores based on the multiplication of positivity and intensity scores, we categorized samples into two groups: high SOX4 expression (immunoreactivity scores ≥ 4) and low SOX4 expression (immunoreactivity scores < 4) in RB tumors (Supplementary Fig. 6 and Table 1). Immunoreactivity analysis revealed that SOX4 was predominantly localized in the cell nucleus, with 20 samples classified as high SOX4-expression (42.6%) and 27 samples as low SOX4-expression (57.4%) RB tumors. Our findings demonstrated a significant correlation between SOX4 expression levels and optic nerve invasion (*P* = 0.0089), while no specific correlations were observed with other clinicopathological features (Supplementary Table 1). These results suggested that *SOX4* may play regulatory roles in the local extension of RB, providing a potential therapeutic target for preventing RB local extension.

## Discussion

The prognosis of RB patients worsens drastically once RB tumor breaches cribriform plate or sclera and develops local extension^9^. Although significant progress has been made in eye-preserving treatment for RB, enucleation is still recommended for RB eyes in the extraocular stage and those in advanced intraocular stage with high-risk histopathological features to prevent life-threatening extraocular extension, recurrence, and metastasis^18–20^. However, the mechanism of local extension of RB has not yet been elucidated. Single-cell sequencing technology had been applied to identify intra-tumoral heterogeneity in RB^13,16^, and trace the cell origin of RB in organoid^21^. Yang et al. delineated the cellular heterogeneity and malignant progression of RB by profiling 14,739 single cells in two RB samples^13^. They presented the first single-cell transcriptomic profile of RB samples. However, there has been no study comparatively investigating tumor cell heterogeneity and molecular characteristics that give rise to RB local extension and metastasis, and the core cell subpopulations and genes contributing to extraocular extension of RB remain unknown. In this study, we performed single-cell transcriptome profiling on four RB samples (two from intraocular RB patients and two from extraocular RB patients) and integrated public scRNA-seq datasets of five normal retina samples, four intraocular, and three extraocular RB samples. We identified nine major cell types in human retinoblastoma, and cone precursor-like cells, retinoma-like cells, and MKI67^+^ PhrD cells constituted the major cell components of both intraocular and extraocular RB samples, which is consistent with the cell heterogeneity found in organoid retinoblastoma^21^. The number of cells in individual samples could bias some results, especially the comparison of cell populations between different groups. As the scRNA-seq protocol has been developed more standardized, this bias has been much reduced. This is acceptable in many scRNA-seq studies^15,22–24^. A larger sample size could lead to a more accurate conclusion. As far as we know, our study presented the single-cell intraocular-extraocular comparison study with the largest sample number up to now. To avoid overstatement, we restricted the statement to the analyzed dataset.

RB tumor progression is reflected by a gradual loss of differentiation and photoreceptor expression signature^25^. In our study, decreased expression of the gene *PDC* (encoding Phosducin, a photoreceptor-specific protein participating in visual phototransduction and photoreceptor metabolism) was observed in all three major cell types in extraocular RB samples, indicating a loss of differentiation and photoreceptor expression signature in extraocular RB tumors, suggesting a higher degree of malignancy. Many of the top differentially expressed genes between intraocular and extraocular RB samples were found in both CPL and MKI67^+^ PhrD cells. The *PEG10* gene (encoding the Paternally Expressed Gene 10 Protein, reported to have a role in cell differentiation and apoptosis) was downregulated in extraocular RB samples in both CPL and MKI67^+^ PhrD cells, indicating a possible loss of differentiation in cells of extraocular RB tumors. Genes including *TUBB2B*, *TMEM14C*, *TMEM14B*, *NEUROG1*, *DEK*, *SOX4*, *PRL*, and *C6orf62* were identified as the top upregulated genes in extraocular RB samples in both CPL and MKI67^+^ PhrD cells. For instance, overexpression of *TUBB2B* and *TMEM14B* has been correlated with poor outcomes in other cancers^26^. DEK is a well-known oncoprotein that participates in the occurrence, progression, and metastasis of various tumors^27,28^. These differentially expressed genes between intraocular and extraocular RB samples in certain cell types might play important roles in RB progression, which requires further experimental validations.

In MKI67^+^ PhrD cell subpopulations, the major portions of the MKI67^+^ PhrD-4 and PhrD-7 subpopulations were detected at the end of the trajectory (Fig. 4g, h). These MKI67^+^ PhrD cell subpopulations may develop towards the end of the RB local extension progression to extraocular sites and might contribute to the local extension of RB. Further experiments are needed to validate the presence of these cell subpopulations and their cellular functions in promoting the progression of RB local extension. This finding also indicates a potential diagnosis and treatment strategy for RB patients. Specifically suppressing these cell subpopulations might be effective in preventing or delaying the local extension progression of RB diseases.

Historical data from numerous studies suggest that approximately 46% (157/340 overall, ranging from 39% to 59%) of RB samples exhibit a 6p gain, with a higher prevalence in extraocular RB samples^29–37^. However, many of these studies lack comprehensive clinicopathologic details. From those providing thorough information, a variable prevalence of 6p gain is observed when comparing extraocular with intraocular RB samples. For instance, Josefina et al. documented a 6p gain in 14 out of 16 extraocular RB samples, compared to none in 17 intraocular RB samples using comparative genomic hybridization^29^. Gustav et al. found a 6p gain in 5 out of 6 extraocular RB samples against 13 out of 36 intraocular RB samples through whole genome sequencing^30^. Both findings underscore the potential link between 6p gain and the local extension of RB. In contrast, another study, including solely intraocular RB cases, identified 6p gains in 28 of the 54 intraocular RB samples using cfDNA sequencing in the aqueous humor^31^. The lack of 6p gains in our intraocular RB cases could be due to our smaller sample size, and variations in detection methods might also be a factor. In our scRNA dataset, which shows a 6p gain in 4 of 5 extraocular samples against none in 6 intraocular samples, there is an implied connection between 6p gains and the localized extension of RB. Our comparative analysis between intraocular and extraocular RB strongly suggests the critical role of 6p amplification in the local extension progression of RB diseases. However, it remains uncertain which gene or genes on 6p drive the aggressive activity of RB^33^. In our study, *SOX4*, located on 6p22.3, exhibited a remarkably higher AUC value of regulatory network inference and a higher expression level in cells from extraocular RB samples. In addition, the regulatory activity of *SOX4* was only detected in MKI67^+^ PhrD cells of extraocular RB samples. Our experiments of qPCR, western blot, and immunofluorescence on additional clinical RB samples further validated that *SOX4* was highly expressed in extraocular RB samples compared to intraocular RB ones. The *SOX4* gene encodes a member of the SOX (SRY-related HMG-box) family of transcription factors involved in the regulation of embryonic development and in the determination of cell fate. *SOX4* is a marker of poor prognosis that contributes to tumor progression and metastasis formation, with aberrantly high expression in a wide variety of aggressive cancers^38–42^, and is known to be a master regulator of epithelial mesenchymal transition^43^. Our results suggest that SOX4 is a potential regulator that might drive RB local extension and could be a target candidate for preventing RB invasive progression.

In conclusion, our study characterized the single-cell transcriptional atlas of intraocular and extraocular RB samples, revealing the differences in cellular and molecular heterogeneity between primary RB and RB with local extension. Our analysis also identified candidate cell subpopulations and TFs that may participate in the progression of RB local extension. These findings offer single-cell insights into the disease progression of RB and provide potential targets for diagnosis and therapy of RB patients.

## Methods

### Collections of clinical specimens

Human eye samples were collected immediately following eye enucleation. Tissue was dissected to isolate the tumor region for single-cell dissociation. Patients with or without involvement of the retrobulbar optic nerve were first screened by imaging diagnosis before surgery and finally validated by pathological diagnosis after surgery. Human tissue samples were obtained with patient informed consent and the approval of the Institutional Review Board at Zhongshan Ophthalmic Center.

### Single-cell collections

Fresh human RB tissue samples were immediately washed three times in ice-cold PBS after collection. Dissociation into single cells was achieved by incubation in 0.25% trypsin-0.01% EDTA (Invitrogen) at 37 °C for 5 min, followed by gentle pipetting and flicking. Trypsin was then blocked with the addition of FBS (Gibco). Dissociated cells were filtered with 70 μm cell strainer (Corning), centrifuged at 1000 rpm for 5 min, and then resuspended in 200 to 500 ul ice-cold PBS. Cell counting and viability rate were determined with Countess II automated cell counter (Invitrogen) after 0.4% trypan blue staining. The sample quality was considered ideal for further single-cell assessment only when the cell viability was higher than 90%, and the clustering rate was less than 1%. Samples were processed within 3 h from surgical removal to loading on the Chromium (10x Genomics) instrument.

### Single-cell RNA sequencing

Gel Bead-In-EMulsions (GEMs) are formed by combining the prepared single-cell suspension with gel beads containing barcode information and a mixture of enzymes. These GEMs are then encapsulated by oil droplets located in microfluidic single-cross junctions. Valid GEMs consist of beads (pre-made 10x primers in beads), single cells, and Master Mix. Subsequently, cell lysis and reverse transcription reactions were performed within the GEMs. In valid GEMs, the 10x Barcode is ligated with the cDNA product. Next, GEMs and oil droplets were broken. PCR amplification was carried out using the cDNA as a template. After the completion of amplification, a quality inspection of the amplified products (evaluating the size of the amplified fragments and the yield of the products) was conducted. Once the amplification products met the quality criteria, the sequencing library was constructed. Initially, the cDNA was chemically broken into fragments of about 200–300 bp. The fragmented cDNA underwent end-repaired and A-tailing. Subsequently, the cDNA fragments were screened, and the P7 adapter was connected, introducing the sample index through PCR amplification. Finally, fragment screening was performed to obtain cDNA library. Once the library was completed and passes the inspection, the Illumina NovaSeq sequencing platform was employed for sequencing to obtain data for subsequent analysis.

### ScRNA-seq data processing

The raw scRNA-seq reads were initially processed using the Cell Ranger (version 6.0.2) software^44^ with default settings. Briefly, the scRNA-seq reads were aligned to the human reference genome (GRCh38) using the splice-aware aligner STAR (version 2.7.9a)^45^. Low-quality cells were excluded based on three metrics^46^. Briefly, data quality in each cell was assessed using the total UMI counts, the number of expressed genes, and the proportion of read counts from mitochondrial genes. Cells were designated as low-quality cells and filtered out if they met any of the following criteria: (1) total UMI counts lower than the median of all cells minus three times the median absolute deviation; (2) expressed genes fewer than the median of all cells minus three times the median absolute deviation; (3) proportion of mitochondrial gene counts higher than the median value of all cells plus three times the median absolute deviation. The filtered data was then utilized in the subsequent analysis.

### Doublet detection and removal

During the library construction process, there is a possibility that two or more cells may enter the same microfluid droplet, resulting in undistinguished barcodes. These occurrences are referred to as doublets or multiplets. To identify these doublets in our scRNA-seq data, the DoubletFinder software (version 2.03)^47^ was employed. In this approach, artificial doublets were first created by calculating the average transcriptional profile of randomly chosen cell pairs. Subsequently, the gene expression of each cell was mapped to the artificial doublets, and doublets were predicted based on the proximity between them.

### Integration of scRNA-seq data from all samples

The Seurat R package (version 4.0.3)^48^ was employed to integrate scRNA-seq data from all cells across the investigated samples. In summary, the datasets were normalized, and the top 2000 variable genes were selected using appropriate thresholds for mean expression and dispersion to facilitate integration. The FindIntegrationAnchors function identified anchors between samples, and these anchors were then used in the IntegrateData function to perform datasets integration. Subsequently, an integrated data assay containing all cells was created for downstream analysis.

### Unsupervised clustering analysis and dimensionality reduction

Before cell clustering, we calculated cell cycle phase scores in each cell using a list of canonical markers^49^. Cell cycle scoring was performed using the CellCycleScoring function implemented in the Seurat package. Gene features in the integrated data assay were first scaled and centered using the ScaleData function. The effects of the cell cycle were regressed out during the scaling process. The RunPCA function was then employed to perform dimensionality reduction through principal component analysis (PCA) with 30 principal components. Subsequently, the Uniform Manifold Approximation and Projection (UMAP) dimensional reduction technique was applied with PCA reduction as input, utilizing the RunUMAP function in the Seurat package. Dimensions from 1 to 30 were used in the reduction procedure. Next, the k.param nearest neighbors were computed by using the FindNeighbors function with PCA reduction and dimensions 1-30. Finally, the FindClusters function was utilized to identify cell clusters through shared nearest neighbor modularity optimization-based unsupervised clustering. The resolution was set to 0.8 to obtain cell communities, and the clustered cells were visualized using the UMAP results.

### Cell type assignment

We compiled known gene markers for various cell types from previous studies^21,50–52^. These included cone precursor-like cells (*CRX*, *RXRG*, *THRB*), MKI67^+^ photoreceptorness decreased cells (*UBE2C, BIRC5, TPX2*, *KIF14*, *MKI67*, *TOP2A*), rod precursor-like cells (*CRX*, *NRL*, *RCVRN*), retinoma-like cells (*CDCA7*, *HELLS*, *MCM3*, *PCNA*), rods or rod-like cells (*CNGA1*, *GNAT1*, *NR2E3*, *NRL*, *PDE6A*, *PDE6G*, *RHO*), bipolar cells (*CA10*, *LRTM1*, *PCP2*, *PRKCA*, *TRPM1*, *VSX1*, *VSX2*), Müller glia (*APOE*, *C1orf61*, *CLU*, *GLUL*, *RLBP1*, *SPP1*, *VIM*), microglia (*AIF1*, *APOE*, *C1QA*, *HLA-DPA1*, *HLA-DRA*, *PTPRC*) and cones or cone-like cells (*ARR3*, *GNB3*, *GNGT2*, *GUCA1C*, *OPN1LW*, *PDE6H*). Then, a semi-supervised machine learning method for cell type assignment was used to map the expression patterns of these marker genes in all cell clusters^53^. Cell clusters with distinguishable expression levels of marker genes were assigned the corresponding cell types. Manual adjustments were made to the assigned cell types based on the expression of corresponding marker genes.

### CNV inference from scRNA-seq data

The InferCNV (version 1.6.0) software (https://github.com/broadinstitute/infercnv) was utilized to infer CNVs in scRNA-seq data following a previously described approach^54^. Initially, CNVs were assessed based on gene expression levels, with genes ordered by their genomic locations. For each chromosome, a sliding window of 100 genes was employed to calculate average expression values. Subsequently, relative CNVs were determined from the outputs generated by InferCNV. Average CNV values were rounded to the nearest integers.

### Different expression analysis

For each cell type or cluster, differentially expressed genes (DEGs) were determined between samples from different groups using the FindMarkers function in the Seurat package. Genes with a fold change > 1.5 and a Benjamini-Hochberg adjusted *p* value < 0.05 were considered as DEGs. Subsequently, these DEGs were analyzed with the clusterProfiler package^55^ for functional enrichment in Gene Ontology biological processes.

### Classification of cell subpopulations

For major cell types, such as cone precursors, retinoma-like cells, and RB cells, we further subdivided them into subpopulations based on distinct gene features. The FindClusters function from the Seurat package was utilized for subpopulation identification in each cell type. Different resolutions were applied to optimize the number of subpopulations for each cell type.

### Pseudo-time trajectory inference

The pseudo-time trajectory was inferred using the Monocle3 (version 1.2.9) software, designed to reconstruct single-cell gene expression kinetics during cellular processes^56^. DEGs between different states were identified using the fit_models function (*Q* value < 0.001). The trajectory was visualized in UMAP graphs, and dynamic expression heatmaps were generated using the Heatmap function in ComplexHeatmap package.

### Single-cell regulatory network inference and clustering

To investigate the dynamic network of regulons, we utilized pySCENIC^17^ (version 0.12) with its standard settings. pySCENIC constructed a co-expression network involving transcription factors and their corresponding target genes. Subsequently, these modules were subjected to motif enrichment through the cisTarget database (hg38_10kbp_up_10kbp_down_full_tx_v10_clust.genes_vs_motifs.rankings.feature and hg38_500bp_up_100bp_down_full_tx_v10_clust.genes_vs_motifs.rankings.feature). Lastly, regulon activity was scored for each individual cell using the AUCell tool.

### Immunofluorescence staining

RB paraffin sections were deparaffinized and rehydrated, followed by antigen retrieval in tris-EDTA buffer (PH 8.0) at 60 °C for 3 h. The sections were then incubated with primary antibodies against SOX4 (Abcam, ab243739, 2 µg/ml) at 4 °C overnight, followed by incubation with Goat anti-Mouse IgG (H + L) Cross-Adsorbed Secondary Antibody, Alexa Fluor™ 488 (Invitrogen, A-11001, 1 µg/ml) for 1 h at room temperature in the absence of light. Nuclei were labeled blue with DAPI (Abcam, ab104139, 2 drops/slide). The images were captured using a confocal microscope (ZEISS LSM 980).

### RNA extraction and quantitative real-time qPCR

Total RNA was extracted using TRIzol (Ambion, Austin, TX, USA) and quantified with Nanodrop 2000 (ThermoFisher Scientific). Complementary DNA (cDNA) was synthesized from 1 μg of RNA using the Reverse-transcription Reagent Kit with gDNA Eraser (Takara, Tokyo, Japan). Samples were incubated at 37 °C for 15 min for reverse transcription, followed by denaturation at 85 °C for 5 s (GeneAmp PCR system 9700 Thermocycler, ABI, Foster, CA). The reverse transcribed cDNA product was used for gene expression analyses, performed using QuantiNova SYBR Green PCR Kit (Qiagen, Duesseldorf, Germany). Primers for *SOX4* (Forward: AGCGACAAGATCCCTTTCATTC, Reverse: CGTTGCCGGACTTCACCTT) and *GAPDH* (Forward: AGGTCGGTGTGAACGGATTTG, Reverse: GGGGTCGTTGATGGCAACA) were obtained from Sangon Biotech, Shanghai, China. Real-time qPCR reaction was carried out using the Roche Light Cycler 480 (Roche, Basel, Switzerland) with an initial denaturation at 95 °C for 2 min, followed by 40 cycles of denaturation at 95 °C for 5 s, annealing and extension at 60 °C for 10 s to collect fluorescent signals. Amplification of *GAPDH* was included for each sample and served as internal control.

### Western blotting

Total protein lysates were extracted from RB tissues to assess the expression level of SOX4. Extracted protein lysates were analyzed using a standard Western blotting protocol for the expression of SOX4 (Abcam, ab70598, 1:500) with GAPDH (Proteintech, 10494-1-AP, 1:10000) as the internal control. After exposing SOX4, the PVDF membrane was treated with an antibody stripping solution, followed by re-incubation with the primary antibody against GAPDH. The primary antibodies for SOX4 and GAPDH were sourced from mouse and rabbit, respectively. The HRP-conjugated Affinipure Goat Anti-Mouse IgG (H + L) (Proteintech, AB_2722565, 1:20000) and the Goat anti-Rabbit IgG (H + L) Secondary Antibody, HRP (Invitrogen, AB_228341, 1:10000) were used.

### Immunohistochemistry

Immunohistochemistry (IHC) was performed on 4-μm-thick paraffin sections of human tissues using standard protocols with optimized conditions. Tissue samples for IHC assays were obtained from a cohort of 47 RB patients who had undergone treatment at the Zhongshan Ophthalmic Center. All RB samples were reviewed by specialized pathologists. The expression of SOX4 (Abcam, ab243041, 1:1000) was evaluated with the HRP-conjugated Goat anti-mouse IgG (H + L) (Servicebio, GB23301, 1:200) using semi-quantitative methods that considered both the proportion and intensity of stained tumor cells. The percentage of positively stained cells was counted in five randomly selected fields under a light microscope (×400), and staining intensity was graded as follows: negative (0), weak (1+), moderate (2+), or intense (3+). The percentage of stained cells was categorized as follows: ≤10% (grade 1), 11–50% (grade 2), or >50% (grade 3). The final IHC scores were calculated by multiplying the scores for the percentage of stained cells and staining intensity. In this study, the highest score obtained was 9, while the lowest was 0. SOX4 expression was deemed high when the IHC score was ≥ 4 and low when the IHC score was < 4. The significance of the difference was evaluated using Fisher’s exact test.

### Statistics and reproducibility

Statistical analysis and data visualization in this study were performed using the R software (R Foundation for Statistical Computing, Vienna, Austria, http://www.r-project.org). Unless specifically stated, all statistical tests were two-tailed, and a *p* value or false discovery rate (FDR) < 0.05 was considered as statistically significant.

### Reporting summary

Further information on research design is available in the Nature Portfolio Reporting Summary linked to this article.

### Supplementary information

Supplementary Information

Description of Additional Supplementary Files

Supplementary Data 1

Supplementary Data 2

Supplementary Data 3

Supplementary Data 4

Reporting Summary

## Data Availability

The scRNA-seq data generated in this study has been deposited in the Gene Expression Omnibus (GEO) database with the accession of GSE249995. All data are available from the corresponding authors on reasonable request. Software and resources used for analysis and plotting are described in each method section. The source data behind the graphs in the manuscript can be found in Supplementary Data 4.

## Code availability

Scripts and codes that were used for data analysis and visualization were deposited in GitHub^57^: https://github.com/lishenglilab /RB_scRNAseq.
