# Supplementary material for: Single-cell transcriptomics enable the characterization of local extension in retinoblastoma
Source: Commun Biol. 2024 Jan 3;7:11. doi: 10.1038/s42003-023-05732-y (PMC10764716; doi:10.1038/s42003-023-05732-y)
Supplement: Supplementary file 1 — Supplementary Information [file 42003_2023_5732_MOESM1_ESM.pdf]

Supplementary Tables

Supplementary Table 1. The association between SOX4 expression and clinicopathological features.

Clinicopathological features	*SOX4* immunoexpression# (n = 47)		P Value*
	High n= 20	Low n= 27	
Sex			
Male (22)	8	14	0.5564
Female (25)	12	13	
Age			
≤ 2 years (19)	8	11	＞0.999
> 2 years (28)	12	16	
Massive Choroidal invasion			
Yes (29)	14	15	0.3739
No (18)	6	12	
Optic nerve invasion			
Yes (27)	16	11	0.0089**
No (20)	4	16	
Iris & AC invasion			
Yes (3)	1	2	＞0.999
No (44)	19	25	
Retinal detachment			
Yes (27)	12	15	＞0.999
No (20)	8	12	
Ki67 expression			
≥ 80% (32)	13	19	0.7583
< 80% (15)	7	8	

Supplementary Figures

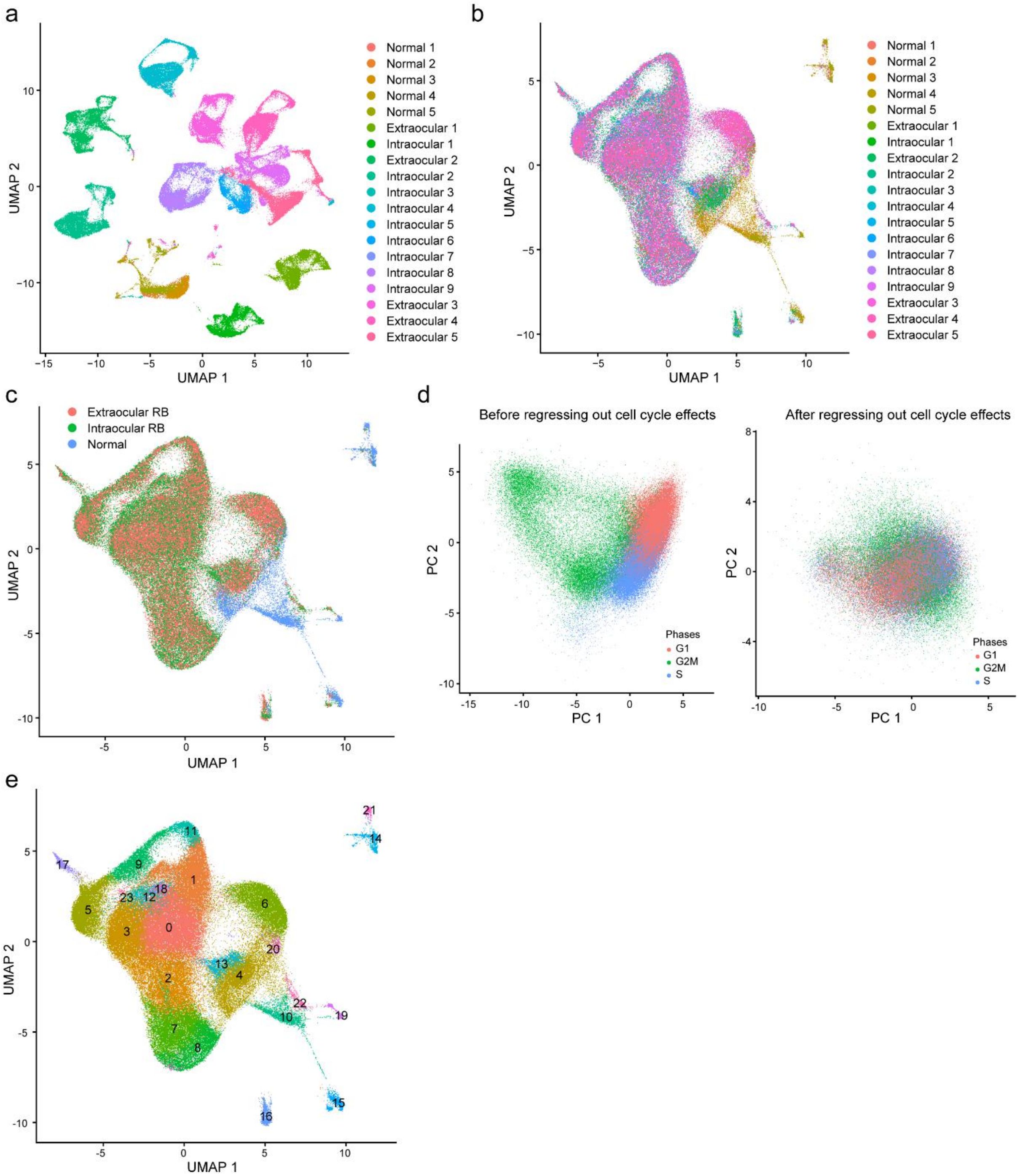

Supplementary Fig. 1 Single-cell transcriptomics in RB samples. a UMAP plot showing all samples before the integration. **b** UMAP plot showing all samples after the integration. **c** UMAP plot shows all cells by different sample groups. **d** PCA analysis of all cells before (left panel) and after (right panel) regressing out the effect of cell cycle based on cell cycle-associated genes. **e** UMAP plot shows cell clusters identified among all cells. In (**a**) and (**b**), Intraocular 3 and Intraocular 4, Intraocular 5 and Intraocular 6, Intraocular 7 and Intraocular 8 are replicates from the same RB patients, respectively.

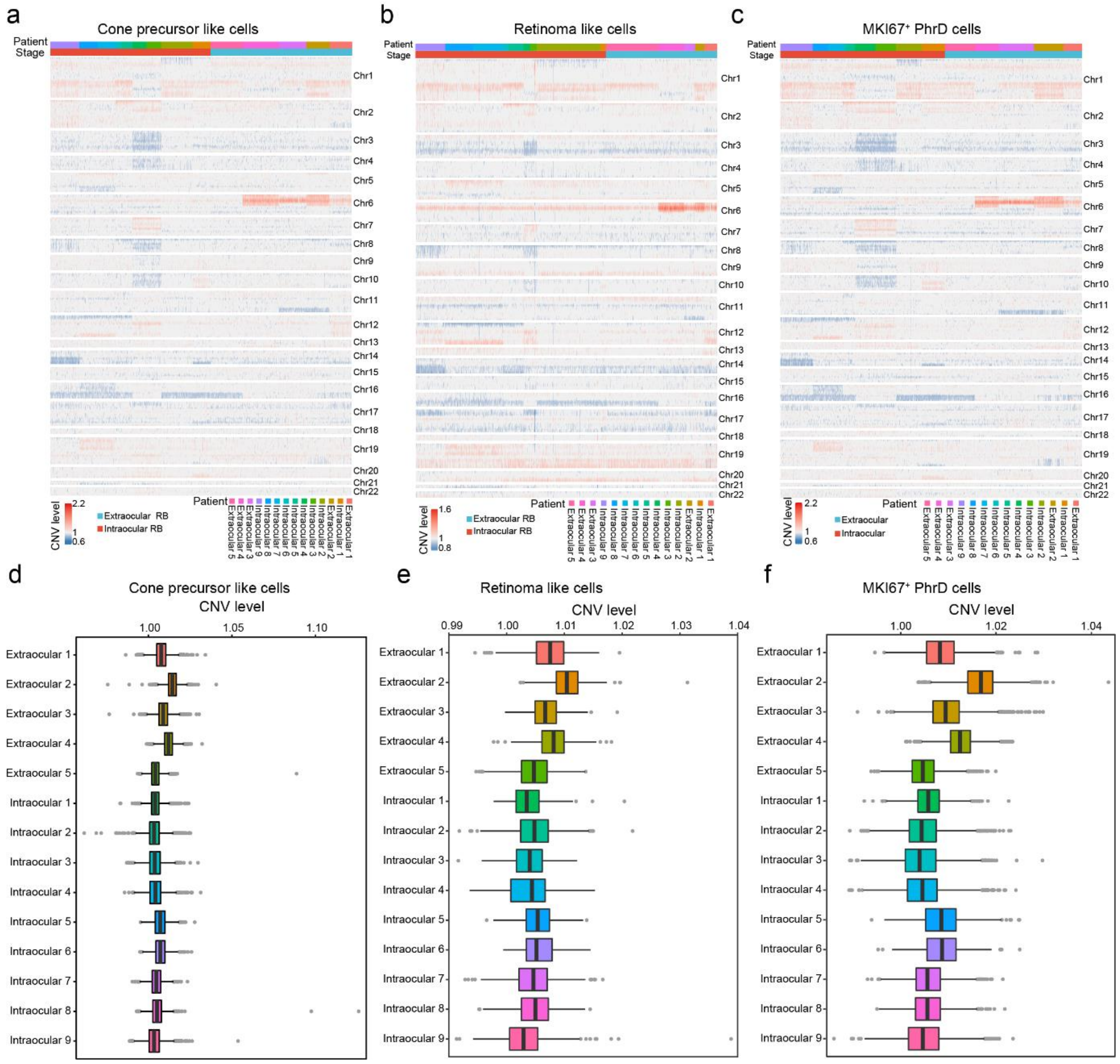

Supplementary Fig. 2 CNV analysis in each RB sample. Heatmaps showing the CNV status of each RB sample across 22 chromosomes in CPL (**a**), RL (**b**), and MKI67^{+} PhrD cells (**c**). Boxplots showing the overall CNV levels of each RB sample in CPL (**d**), RL (**e**), and MKI67^{+} PhrD cells (**f**). In (**a-f**), Intraocular 3 and Intraocular 4, Intraocular 5 and Intraocular 6, Intraocular 7 and Intraocular 8 are replicates from the same RB patients, respectively. In (**d-f**), Each box represents the IQR and median of CNV levels in each sample group, whiskers indicate 1.5 times IQR.

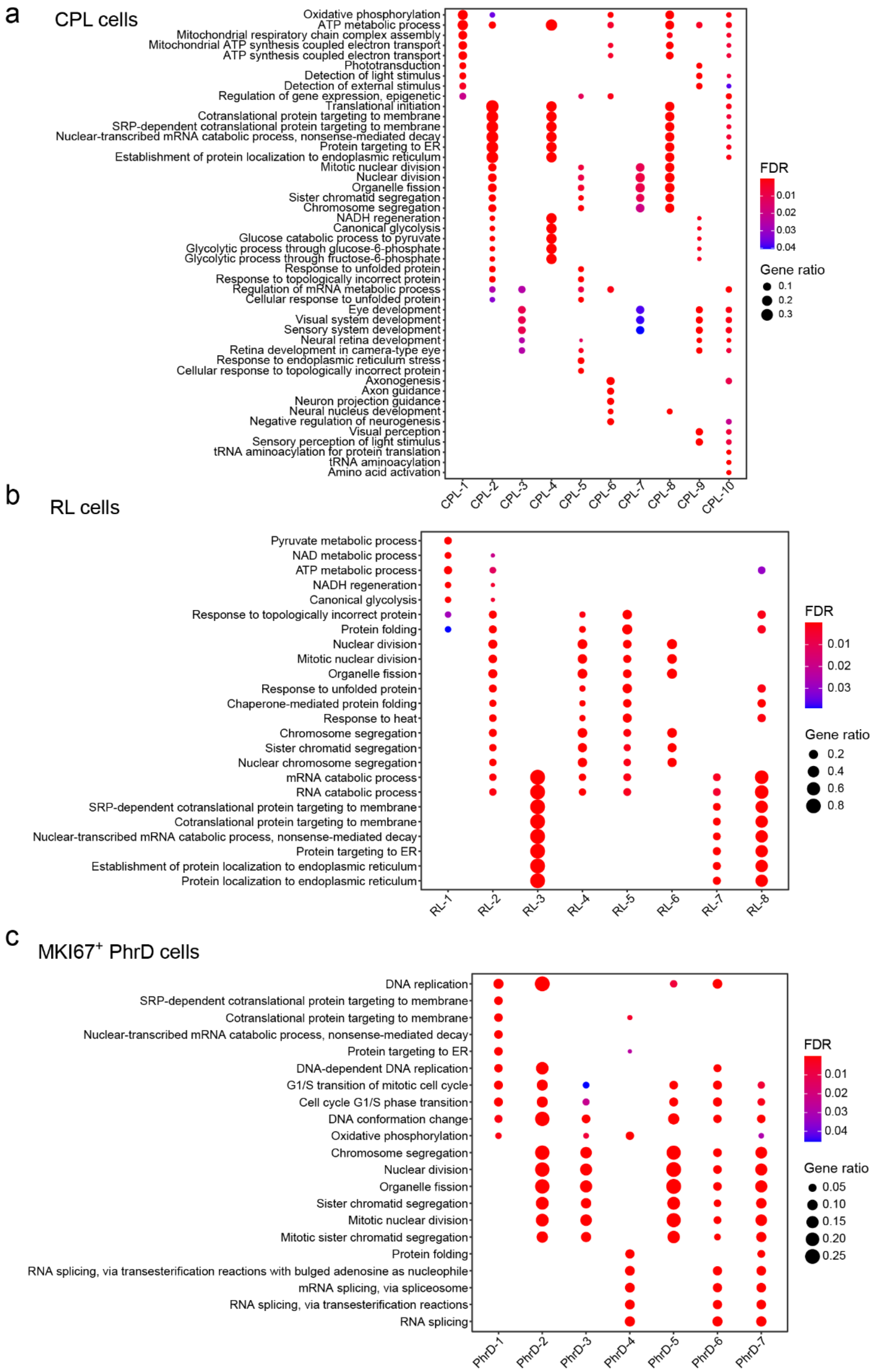

Supplementary Fig. 3 Functional enrichment analysis of differentially expressed genes in Gene Ontology biology processes. Functional enrichment analysis of differential genes of each subpopulation in CPL cells (**a**), RL cells (**b**), and MKI67^{+} PhrD cells (**c**).

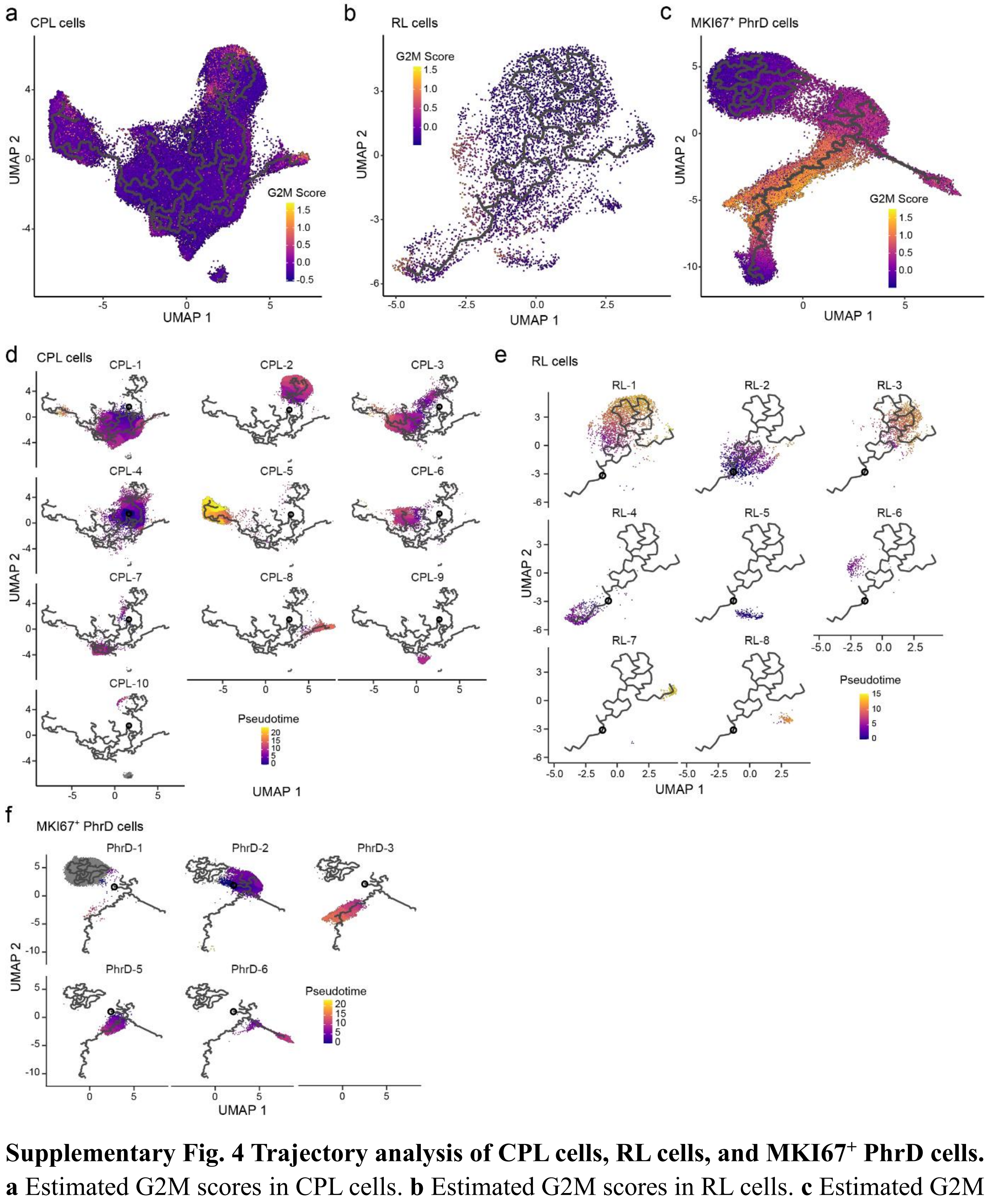

Supplementary Fig. 4 Trajectory analysis of CPL cells, RL cells, and MKI67^{+} PhrD cells. **a** Estimated G2M scores in CPL cells. **b** Estimated G2M scores in RL cells. **c** Estimated G2M scores in MKI67^{+} PhrD cells. **d** The distribution of cells of CPL subpopulations along the pseudo-time path. **e** The distribution of cells of RL subpopulations along the pseudo-time path. **f** The distribution of cells of MKI67^{+} PhrD subpopulations along the pseudo-time path.

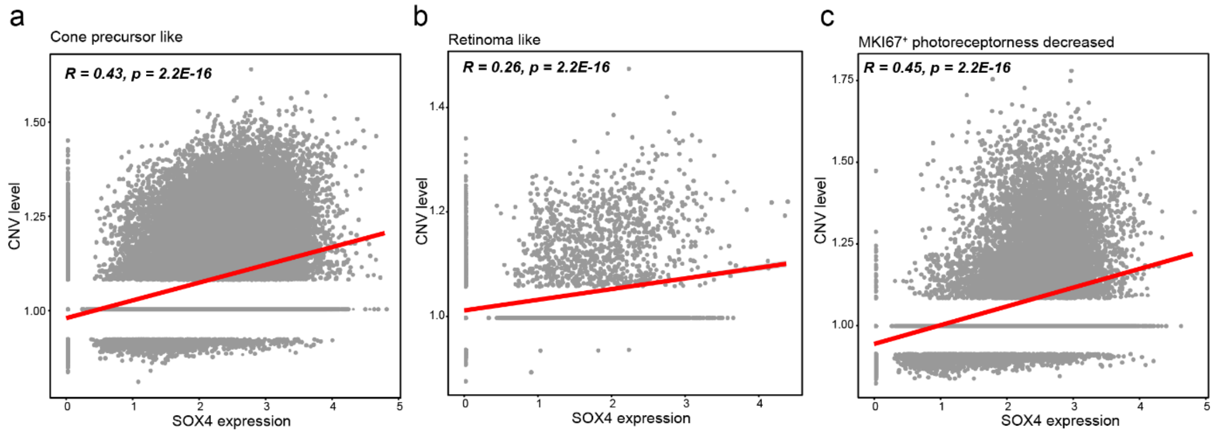

Supplementary Fig. 5 The correlations between SOX4 gene expression and CNV level in cone precursor like cells (a), retinoma like cells (b), and MKI67^{+} photoreceptorness decreased cells (c).

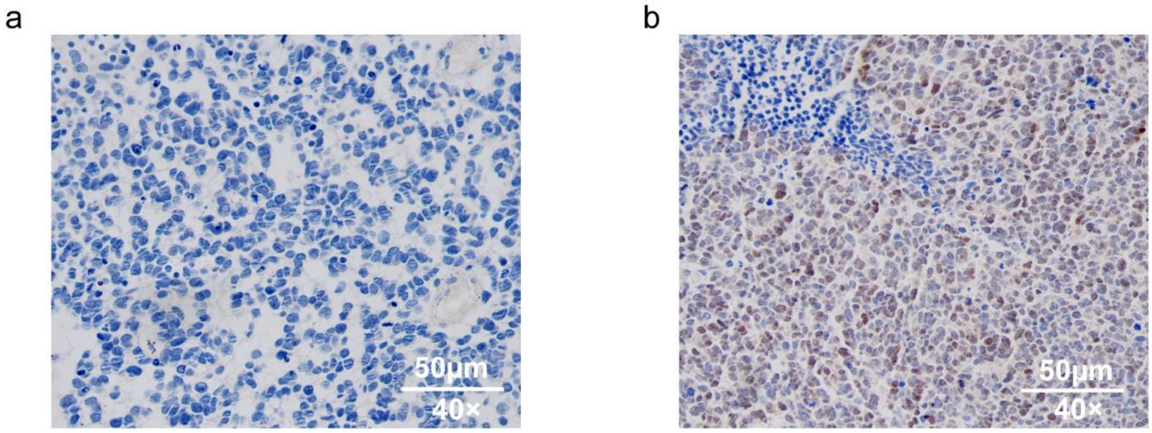

Supplementary Fig. 6 Representative IHC images of SOX4. Representative IHC images of SOX4-low sample (**a**) and SOX4-high sample (**b**).

Fig 5g

Anti-SOX4

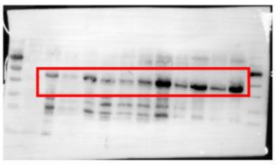

Fig 5g

Anti-GAPDH

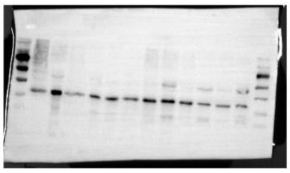

Supplementary Fig. 7 Original gels for Fig. 5. The original western blot of SOX4 and GAPDH for Fig. 5g.
