# Supplementary material for: Single-cell transcriptomics enable the characterization of local extension in retinoblastoma
Source: Commun Biol. 2024 Jan 3;7:11. doi: 10.1038/s42003-023-05732-y (PMC10764716; doi:10.1038/s42003-023-05732-y)
Supplement: Supplementary file 2 — Description of Additional Supplementary Files [file 42003_2023_5732_MOESM2_ESM.pdf]

Description of Additional Supplementary Files

File name: Supplementary Data 1
Description: Top 100 marker genes in each cell type.

File name: Supplementary Data 2
Description: Differentially expressed genes between intraocular and extraocular RB samples in CPL cells, RL cells, and MKI67+ PhrD cells.

File name: Supplementary Data 3
Description: Top 100 marker genes in different cell subpopulations.

File name: Supplementary Data 4
Description: Source data behind the graphs in the figures.
