# Supplementary material for: Single-cell transcriptomics enable the characterization of local extension in retinoblastoma
Source: Commun Biol. 2024 Jan 3;7:11. doi: 10.1038/s42003-023-05732-y (PMC10764716; doi:10.1038/s42003-023-05732-y)
Supplement: Supplementary file 7 — Reporting Summary [file 42003_2023_5732_MOESM7_ESM.pdf]

nature portfolio

Corresponding author(s): Shengli Li
Last updated by author(s): Dec 1, 2023

Reporting Summary

Nature Portfolio wishes to improve the reproducibility of the work that we publish. This form provides structure for consistency and transparency in reporting. For further information on Nature Portfolio policies, see our Editorial Policies and the Editorial Policy Checklist.

Statistics

For all statistical analyses, confirm that the following items are present in the figure legend, table legend, main text, or Methods section.

n/a	Confirmed	
☐	☒	The exact sample size (*n*) for each experimental group/condition, given as a discrete number and unit of measurement
☐	☒	A statement on whether measurements were taken from distinct samples or whether the same sample was measured repeatedly
☐	☒	The statistical test(s) used AND whether they are one- or two-sided<br>*Only common tests should be described solely by name; describe more complex techniques in the Methods section.*
☐	☒	A description of all covariates tested
☐	☒	A description of any assumptions or corrections, such as tests of normality and adjustment for multiple comparisons
☐	☒	A full description of the statistical parameters including central tendency (e.g. means) or other basic estimates (e.g. regression coefficient) AND variation (e.g. standard deviation) or associated estimates of uncertainty (e.g. confidence intervals)
☐	☒	For null hypothesis testing, the test statistic (e.g. *F*, *t*, *r*) with confidence intervals, effect sizes, degrees of freedom and *P* value noted<br>*Give P values as exact values whenever suitable.*
☒	☐	For Bayesian analysis, information on the choice of priors and Markov chain Monte Carlo settings
☒	☐	For hierarchical and complex designs, identification of the appropriate level for tests and full reporting of outcomes
☐	☒	Estimates of effect sizes (e.g. Cohen's *d*, Pearson's *r*), indicating how they were calculated

Our web collection on statistics for biologists contains articles on many of the points above.

Software and code

Policy information about availability of computer code

Data collection	No software was used for data collection.
Data analysis	There is no custom software in this study.

For manuscripts utilizing custom algorithms or software that are central to the research but not yet described in published literature, software must be made available to editors and reviewers. We strongly encourage code deposition in a community repository (e.g. GitHub). See the Nature Portfolio guidelines for submitting code & software for further information.

Data

Policy information about availability of data

All manuscripts must include a data availability statement. This statement should provide the following information, where applicable:

- Accession codes, unique identifiers, or web links for publicly available datasets
- A description of any restrictions on data availability
- For clinical datasets or third party data, please ensure that the statement adheres to our policy

ScRNA-seq data generated in this study has been deposited in the Gene Expression Omnibus (GEO) database. All data are available from the corresponding authors on reasonable request. Software and resources used for analysis and plotting are described in each method section.

Research involving human participants, their data, or biological material

Policy information about studies with human participants or human data. See also policy information about sex, gender (identity/presentation), and sexual orientation and race, ethnicity and racism.

Reporting on sex and gender	Not applicable.
Reporting on race, ethnicity, or other socially relevant groupings	Not applicable.
Population characteristics	Not applicable.
Recruitment	Not applicable.
Ethics oversight	Not applicable.

Note that full information on the approval of the study protocol must also be provided in the manuscript.

Field-specific reporting

Please select the one below that is the best fit for your research. If you are not sure, read the appropriate sections before making your selection.

☒ Life sciences ☐ Behavioural & social sciences ☐ Ecological, evolutionary & environmental sciences

For a reference copy of the document with all sections, see nature.com/documents/nr-reporting-summary-flat.pdf

Life sciences study design

All studies must disclose on these points even when the disclosure is negative.

Sample size	We perform single-cell RNA sequencing (scRNA-seq) on 4 RB samples (2 from intraocular and 2 from extraocular RB patients), and integrate public scRNA-seq datasets of 5 normal retina, 4 intraocular, and 3 extraocular RB samples to characterize the invasive progression of RB at single-cell level. We conducted immunofluorescence of SOX4 in 7 clinical samples diagnosed with intraocular RB and 7 samples diagnosed with extraocular RB. We also performed SOX4 immunostaining in a cohort of 47 RB tumor samples.
Data exclusions	Not applicable.
Replication	All data analyses are reproducible.
Randomization	Not applicable.
Blinding	Not applicable.

Reporting for specific materials, systems and methods

We require information from authors about some types of materials, experimental systems and methods used in many studies. Here, indicate whether each material, system or method listed is relevant to your study. If you are not sure if a list item applies to your research, read the appropriate section before selecting a response.

Materials & experimental systems

n/a	Involved in the study	
☐	☒	Antibodies
☒	☐	Eukaryotic cell lines
☒	☐	Palaeontology and archaeology
☒	☐	Animals and other organisms
☒	☐	Clinical data
☒	☐	Dual use research of concern
☒	☐	Plants

Methods

n/a	Involved in the study	
☒	☐	ChIP-seq
☒	☐	Flow cytometry
☒	☐	MRI-based neuroimaging

Antibodies

Antibodies used	SOX4 (Abcam,ab243739); SOX4 (Abcam, ab70598); SOX4 (Abcam, ab243041); IgG (Invitrogen, A-11001); IgG (Servicebio, GB23301);

Antibodies used

IgG (Proteintech, 10494-1-AP); GAPDH (Proteintech, 10494-1-AP)

Validation

Immunofluorescence analysis of SH-SY5Y (Human neuroblastoma cell line from bone marrow). PFA fixed, Triton X-100 permeabilized SH-SY5Y cells labeling SOX4 using ab243739 at 10 µg/ml (green) in ICC/IF. For western bolot (ab70598), use at an assay dependent concentration. Detects a band of approximately 38 kDa (predicted molecular weight: 47 kDa). Immunohistochemistry (Formalin/PFA-fixed paraffin-embedded sections) analysis of human liver tissue labelling SOX4 with ab243041 at 1/1000 dilution. Heat mediated antigen retrieval performed with citrate buffer pH 6 before commencing with IHC staining protocol.

Plants

Seed stocks

Report on the source of all seed stocks or other plant material used. If applicable, state the seed stock centre and catalogue number. If plant specimens were collected from the field, describe the collection location, date and sampling procedures.

Novel plant genotypes

Describe the methods by which all novel plant genotypes were produced. This includes those generated by transgenic approaches, gene editing, chemical/radiation-based mutagenesis and hybridization. For transgenic lines, describe the transformation method, the number of independent lines analyzed and the generation upon which experiments were performed. For gene-edited lines, describe the editor used, the endogenous sequence targeted for editing, the targeting guide RNA sequence (if applicable) and how the editor was applied.

Authentication

Describe any authentication procedures for each seed stock used or novel genotype generated. Describe any experiments used to assess the effect of a mutation and, where applicable, how potential secondary effects (e.g. second site T-DNA insertions, mosiacism, off-target gene editing) were examined.
